# Planning analysis for locally advanced lung cancer: dosimetric and efficiency comparisons between intensity-modulated radiotherapy (IMRT), single-arc/partial-arc volumetric modulated arc therapy (SA/PA-VMAT)

**DOI:** 10.1186/1748-717X-6-140

**Published:** 2011-10-21

**Authors:** Xiaoqin Jiang, Tao Li, Yongmei Liu, Lin Zhou, Yong Xu, Xiaojuan Zhou, Youling Gong

**Affiliations:** 1 Radiation Physics Center, Cancer Center, West China Hospital, Sichuan University, Chengdu, Sichuan Province, PR.China; 2 Department of Thoracic Oncology and Radiation Oncology, Cancer Center, West China Hospital, Sichuan University, Chengdu, Sichuan Province, PR.China; 3 State Key Laboratory of Biotherapy, West China Hospital, Sichuan University, Chengdu, Sichuan Province, PR.China

**Keywords:** Lung cancer, Intensity-modulated radiotherapy, Volumetric modulated arc therapy, Target dose distribution, Normal tissue toxicity

## Abstract

**Purpose:**

To analyze the differences between the intensity-modulated radiotherapy (IMRT), single/partial-arc volumetric modulated arc therapy (SA/PA-VMAT) techniques in treatment planning for locally advanced lung cancer.

**Materials and methods:**

12 patients were retrospectively studied. In each patient's case, several parameters were analyzed based on the dose-volume histograms (DVH) of the IMRT, SA/PA-VMAT plans respectively. Also, each plan was delivered to a phantom for time comparison.

**Results:**

The SA-VMAT plans showed the superior target dose coverage, although the minimum/mean/maximum doses to the target were similar. For the total and contralateral lungs, the higher V_5/10_, lower V_20/30 _and mean lung dose (MLD) were observed in the SA/PA-VMAT plans (*p *< 0.05, respectively). The PA-VMAT technique improves the dose sparing (V_20_, V_30 _and MLD) of the controlateral lung more notably, comparing to those parameters of the IMRT and SA-VMAT plans respectively. The delivered monitor units (MUs) and treatment times were reduced significantly with VMAT plans, especially PA-VMAT plans (for MUs: mean 458.3 *vs*. 439.2 *vs*. 435.7 MUs, *p *< 0.05 and for treatment time: mean 13.7 *vs*. 10.6 *vs*. 6.4 minutes, *p *< 0.01).

**Conclusions:**

The SA-VMAT technique achieves highly conformal dose distribution to the target. Comparing to the IMRT plans, the higher V_5/10_, lower V_20/30 _and MLD were observed in the total and contralateral lungs in the VMAT plans, especially in the PA-VMAT plans. The SA/PA-VMAT plans also reduced treatment time with more efficient dose delivering. But the clinical benefit of the VMAT technique for locally advanced lung cancer needs further investigations.

## Introduction

VMAT is a new form of IMRT, which allows irradiation with the simultaneously changing gantry position, dose rate and multileaf-collimator (MLC) position [1]. Recently, several studies have been published showing the potential of the VMAT techniques to reduce the treatment time without compromising plan quality compared to IMRT in radiotherapy planning for different cancer types, including lung cancers [2-6], prostate cancer [7] and annal cancer [8]. In practice, the VMAT optimization depends on the choice of various plan parameters, such as the number of arcs, the delivery time or the gantry angle spacing between subsequent control points.

A few studies investigated the clinical and dosimetric advantages of the VMAT technique in treatment for lung cancer presently. Bedford *et al*. reported the first lung cancer case treated with VMAT [2]. Scorsetti *et al*. reported the acute toxicity, initial outcome results and planning parameters of 24 patients with the large-volume non-small cell lung cancer (NSCLC) treated with VMAT, while no dosimetric comparison was described [3]. For peripheral lung cancers, McGrath *et al*. concluded that VMAT allows delivering of the hypofractionated doses much faster than the conventional stereotactic body radiotherapy (SBRT), with the additional advantage for the target dose conformity [4]. Cao *et al*. compared the plan quality of intensity-modulated arc therapy (IMAT) and helical tomotherapy, and stated that IMAT can provide the plan qualities comparable to that of the helical tomotherapy for most cases [5]. Very recently, Holt's team found that a coplanar VMAT for SBRT for early-stage lung cancer achieved a plan quality and skin dose levels better than those coplanar IMRT plans and reduced treatment time at most by 70% [6]. To our knowledge, no dosimetric evaluation had been reported between IMRT and VMAT for locally advanced lung cancer yet.

Up to now, the issues existed that whether or not a single arc VMAT could achieve dose distributions comparable to IMRT plan. One study stated that two or more arcs are required in treatment of the complex-shaped target volumes [9], whereas Bertelsen *et al*. [10] found that a single arc is sufficient to achieve plan quality similar to IMRT. Also, data from Guckenberger *et al*. indicated the complexity of the target volume determined whether single arc VMAT was equivalent to IMRT [11]. In addition, McGrath's study showed the significant lung dose reduction in VMAT plans appling a partial arc range of 180° coincide with tumor location [4]. As it could improve lung dose sparing and reducing treatment time, the possible and potential benefits of PA-VMAT in treatment for central and bulky lung cancer are not clear so far.

Here, we reported our planning analysis for the locally advanced lung cancers in these two fields: comparing the dosimetric parameters derived from IMRT and SA/PA-VMAT plans and evaluating treatment delivery efficiency and treatment times.

## Materials and methods

This study was conducted between October 2010 and March 2011. Totally, 12 patients with pathologically confirmed locally advanced NSCLC were randomly selected for analysis. The patient characteristics were listed in Table 1. All patients were staged according to the modified 1997 AJCC staging system [12]. Permission to conduct the study was granted by the Research Ethics Board of the University Health Network.

**Table 1:** Basic and clinical characteristics of the studied patients (n = 12).

***Age (years)***	
Median	53
Range	39-64
***Sex***	
Male	10
Female	2
***Pathology***	
Non-small cell lung cancer	12
***Disease stage***	
IIIa	3
IIIb	9
***PTV volume (cm^3^)***	
Median	221.3
Range	177.6-412.5
***Total lung volume (cm^3^)***	
Median	3525.7
Range	2676.2-4810.5

### Target delineation and dose prescription

Patients were simulated supine in an individualized thermoplastic mask with their arms raised above the head on a lung board designed to support the elbows. All of the computed tomograph (CT) images (Siemens, Somatom Plus 4) of the patients were transferred to and registered in the treatment planning system (TPS).

The gross tumor volume (GTV) was defined as the macroscopically identifiable tumor including lymph nodes with a diameter more than 1 cm on CT. The clinical tumor volume (CTV) enclosed the GTV with an 8 mm margin towards lung tissue and a 5 mm margin around affected lymph nodes. For the planning target volume (PTV), the 10 or 15 mm margin was added isotropically to the CTV if the tumor location is superior or inferior lobe. The organs at-risk (OARs) included: lungs (ipsilateral and contralateral), spinal cord, esophagus and heart. The planning organ at-risk volumes (PRVs) were extended as 5 mm to the esophagus and the spinal cord, respectively; all expansion was not applied to the lungs. The dose-volume constraints for the lungs were set as follows: V_20 _< 30%, V_30 _< 20% and mean lung dose < 20 Gy. The maximum dose of 45 Gy was allowed to the spinal cord (PRV). In addition, the plan optimization was performed with the aim to keep the esophagus PRV dose of 55 Gy (V_55_) to 30% of the organ volume and the heart PRV dose of 40 Gy (V_40_) to 50% of the organ volume.

All generated plans for each patient consisted of 68 Gy to be delivered to PTV in 34 fractions. The objective of planning was to deliver the prescribed dose to at least 95% of the PTV with a dose range not exceeding -10% and +15% of the prescribed dose. All plans were generated for the Elekta Beam Modulator (Elekta Oncology Systems, Crawley, UK).

### Treatment planning and optimizing

#### IMRT

**Figure 1:**
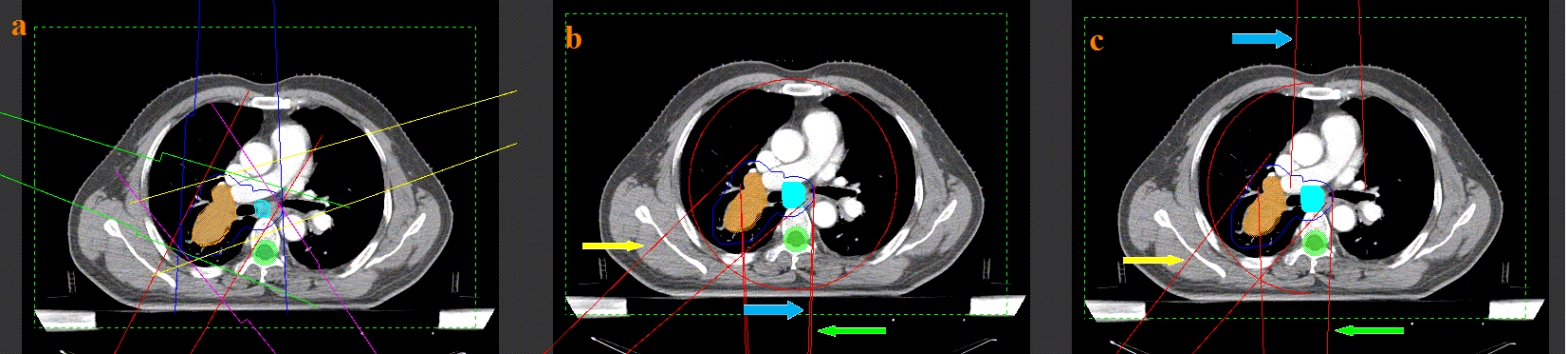
**Representative beam arrangements (a: IMRT, b: SA-VMAT and c: PA-VMAT)**. The green, blue and yellow arrows indicate the start point, end point and one control point in SA/PA-VMAT plans, respectively.

The IMRT optimization was performed by appling Direct Machine Parameter Optimization (DMPO) algorithm in our treatment planning system (Pinnacle^3^, Philips Radiation Oncology Systems, Fitchburg, USA) as described previously [13]. For each plan, an average of 40 segments were used based on 5 (whose angels were 216°, 288°, 0°, 72°, 144°, respectively) or 7 (whose angels were 204°, 256°, 308°, 0°, 52°, 104°, 156°, respectively) coplanar beams with the angles depending on the tumor location (Figure 1a). In the plan generation, the maximum iterations in the plan optimizing were 40, and the maximum number of all segments in one plan was restricted to 100. There is no limitation to the MUs per segment. Plans were generated for the Elekta Beam Modulator with 6-MV.

#### SA-VMAT

The VMAT planning was done applying the SmartArc planning algorithm in Pinnacle^3® ^9.0 (research version, Philips Radiation Oncology Systems, Fitchburg, USA). The optimiser (single arc) was constrained to use one single 360° arc which consisted of 90 control points. The arc was represented by 89 beams each separated by 4° (Figure 1b), which started and ended at 180°, respectively. The accelerator used automatic dose rate selection which ensures that the maximal possible dose rate was chosen for each individual segment of the arc. The initial step is performed based on SmartArc algorithm to obtain the optimal modulated fluence. In the second step, the segments are optimized based on the small ares of targets with insuffient irradiation dose using the same algorithm. Plans were generated with 6-MV either.

#### PA-VMAT

The plans were optimized in the same planning system mentioned above. A 180-200° partial arc was generated for standardization across the studied cases with the range coinciding with the tumor location while avoiding as much of the contralateral lung as possible, which started between 170°-180° and ended between 0°-10°. The arc was represented by 44-49 beams each separated by 4° either (Figure 1c).

### Evaluation of the DVH-based parameters

The conformity index (CI) and homogeneous index (HI) for PTV was calculated as we described previously [13]. The CI was defined as cover factor (the percentage of the PTV volume receiving 68 Gy) ×spill factor (the volume of the PTV receiving the 68 Gy relative to the total prescription dose-volume). The HI was defined the minimum dose in 5% of the PTV (D_5_)/munimum dose in 95% of the PTV (D_95_).

Other parameters were collected from the DVH of these generated plans and compared to each other respectively, including V_5/10/20/30 _(the percentage volumes which received 5, 10, 20 and 30 Gy respectively) of lungs, average dose (D_mean_) of lungs/heart/esophagus, V_30 _and V_40 _(the percentage volumes which received 30 and 40 Gy respectively) of heart, D_max _and D_5 _(the maximum dose and the dose that 5% volume of spinal cord received) of spinal cord and V_55 _(the percentage volumes which received 55 Gy) of esophagus.

All plans were transferred to our treatment system for time comparison.

### Statistical analysis

The collected data were analyzed applying "mean ± standard deviation (SD)" with SPSS software (version 13.0, Chicago, USA). Based on the Wilcoxon's signed rank test, a value of *p *< 0.05 was considered to have statistical significance.

## Results

**Figure 2:**
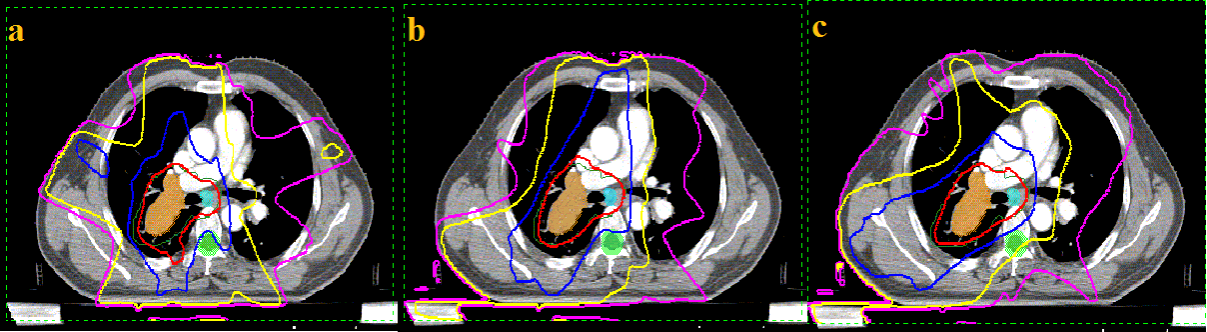
**Transverse sections of the representative plans of one patient with the irradiation isodose curves (a: IMRT, b: SA-VMAT and c: PA-VMAT)**. The pink, yellow, blue and red lines represent the dose curves of 10, 20, 40 and 64.6 (95% of the prescription dose) Gy, respectively.

Totally, 36 plans are generated following the protocol and analyzed. The transverse sections of the representative plans with irradiation dose curves of one patient are shown in Figure 2. Obviously, lower dose irradiated to the larger volume of the total lungs were observed in SA/PA-VMAT plans, respectively.

The evaluation of the DVH-based parameters of the PTV is shown in Table 2. The maximum, minimum and average dose of the PTV are similar between IMRT and SA/PA-VMAT plans respectively, with no statistical significance (*p *> 0.05). While, the average CI in SA-VMAT plans (0.68 ± 0.06) is significantly better than those indexes in plans of IMRT (0.62 ± 0.07) and PA-VMAT (0.62 ± 0.06), respectively (*p *< 0.05). Also, the average HI in SA-VMAT plans (1.11 ± 0.04) is better than those in IMRT (1.15 ± 0.04) and PA-VMAT plans (1.15 ± 0.04) respectively, with the statistical significances (*p *< 0.05).

**Table 2:** Comparisons of the DVH-based parameters of the PTV in present study (n = 12).

	IMRT	SA-VMAT		PA-VMAT		
	mean ± SD	mean ± SD	*p *value*^a^*	mean ± SD	*p *value*^b^*	*p *value*^c^*
D_min_*^d^* (Gy)	62.4 ± 1.5	63.2 ± 1.3	0.179	62.5 ± 1.4	0.908	0.217
D_max_*^d ^*(Gy)	73.9 ± 1.6	73.4 ± 1.5	0.492	73.8 ± 1.6	0.919	0.555
D_mean_*^d ^*(Gy)	68.9 ± 1.5	68.9 ± 1.4	0.949	68.8 ± 1.5	0.840	0.783
CI*^e^*	0.62 ± 0.07	0.68 ± 0.06	0.019	0.62 ± 0.06	0.819	0.025
HI*^f^*	1.15 ± 0.04	1.11 ± 0.04	0.027	1.15 ± 0.04	0.727	0.043

*^a^*: compared with the parameters of IMRT; *^b^*: compared with the parameters of IMRT; *^c^*: compared with the parameters of SA-VMAT; *^d^*: the minimum, maximum and mean irradiation dose of the PTV, respectively; *^e^*: conformity index, calculated with the fomula as we applied previously [13]; *^f^*: homogeneous index, calculated with the fomula: "HI = D_5_/D_95_";

Table 3 shows the comparisons of the DVH-based parameters of the lungs in the present study. Comparing to the IMRT plans, the SA/PA-VMAT plans show advantages in dose sparing of the total and controlateral lungs, respectively (all *p *< 0.05). But IMRT plans show the lower V_5 _and V_10 _of the total (50.6 ± 10.6% and 40.2 ± 7.2%) and contralateral lungs (44.7 ± 10.7% and 32.1 ± 7.1%), comparing to the SA/PA-VMAT plans respectively (all *p *< 0.05). Although the SA/PA-VMAT plans showing a trend to increaseing the volume received irradiation, no siginificant difference was observed in the comparisons between the parameters of the ipsilateral lungs in this study (all *p *> 0.05). Especially, the PA-VMAT technique reduced the V_20 _(7.5 ± 2.2), V_30 _(1.3 ± 0.6) and MLD (7.8 ± 1.0) of the contralateral lungs significantly, comparing to those parameters of the SA-VMAT plans (*p *= 0.049, 0.048 and 0.038, respectively).

**Table 3:** Comparisons of the DVH-based parameters of the OARs^*a *^in present study (n = 12).

	IMRT	SA-VMAT		PA-VMAT		
	mean ± SD	mean ± SD	*p *value*^b^*	mean ± SD	*p *value*^c^*	*p *value*^d^*
***Total lungs***						
V_5_*^e ^*(%)	50.6 ± 10.6	59.7 ± 10.8	0.048	59.3 ± 9.1	0.042	0.923
V_10_*^e ^*(%)	40.2 ± 7.2	46.9 ± 8.3	0.046	46.4 ± 7.3	0.048	0.877
V_20_*^e ^*(%)	25.7 ± 5.7	21.1 ± 4.8	0.044	20.3 ± 4.4	0.016	0.675
V_30_*^e ^*(%)	15.8 ± 3.4	12.9 ± 3.2	0.043	12.5 ± 3.2	0.023	0.876
MLD*^f ^*(Gy)	14.4 ± 2.3	12.5 ± 2.2	0.047	12.3 ± 2.2	0.028	0.785
***Contralateral lungs***						
V_5 _(%)	44.7 ± 10.7	55.2 ± 11.9	0.033	53.8 ± 9.4	0.038	0.752
V_10 _(%)	32.1 ± 7.1	39.4 ± 9.8	0.048	38.5 ± 7.3	0.041	0.801
V_20 _(%)	13.8 ± 4.4	10.2 ± 4.1	0.046	7.5 ± 2.2	< 0.01	0.048
V_30 _(%)	3.3 ± 1.9	2.0 ± 1.0	0.048	1.3 ± 0.6	< 0.01	0.049
MLD (Gy)	9.6 ± 1.2	8.7 ± 1.0	0.038	7.8 ± 1.0	< 0.01	0.038
***Ipsilateral lungs***						
V_5 _(%)	67.6 ± 10.5	68.9 ± 11.8	0.778	72.4 ± 13.7	0.346	0.052
V_10 _(%)	56.8 ± 9.1	57.3 ± 9.7	0.898	61.1 ± 11.5	0.321	0.167
V_20 _(%)	43.9 ± 5.8	45.2 ± 6.3	0.604	43.1 ± 5.2	0.725	0.383
V_30 _(%)	29.8 ± 1.2	29.9 ± 1.4	0.853	29.5 ± 1.2	0.547	0.460
MLD (Gy)	18.8 ± 2.98	20.2 ± 2.9	0.264	18.3 ± 2.8	0.728	0.141
***Spinal cord***						
D_max_*^f ^*(Gy)	43.0 ± 2.5	41.6 ± 2.5	0.194	42.5 ± 2.4	0.603	0.404
D_5_*^g ^*(Gy)	40.3 ± 3.8	39.8 ± 3.9	0.711	40.1 ± 3.9	0.891	0.837
***Heart***						
V_30_*^e ^*(%)	20.9 ± 5.7	20.8 ± 5.8	0.973	21.2 ± 6.3	0.894	0.870
V_40_*^e ^*(%)	14.6 ± 5.5	13.4 ± 5.5	0.595	15.0 ± 5.9	0.859	0.788
D_mean_*^f ^*(Gy)	14.8 ± 7.7	14.4 ± 7.4	0.915	15.1 ± 7.5	0.903	0.818
***Esophagus***						
V_55_*^e ^*(%)	12.4 ± 7.3	12.3 ± 7.5	0.971	13.6 ± 8.3	0.707	0.685
D_mean_*^f ^*(Gy)	22.4 ± 5.6	21.9 ± 5.2	0.811	23.7 ± 6.3	0.609	0.455

*^a^*: organs at-risk; *^b^*: compared with the parameters of IMRT; *^c^*: compared with the parameters of IMRT; *^d^*: compared with the parameters of SA-VMAT; *^e^*: the volume of the lung that received the 5, 10, 20, 30, 40 and 55 Gy irradiation dose, respectively; *^f^*: the maximum or mean irradiation dose that the lung, spinal cord, heart and esophagus received, respectively; *^g^*: the irradiation dose that the 5% volume of the spinal cord received.

The comparisons of the parameters of other OARs in present study are also shown in Table 3. The SA/PA-VMAT techniques do not show advantages in dose sparing of the other evaluating OARs (D_max _and D_5 _to the spinal cord; V_30_, V_40 _and D_mean _to the heart; V_55 _and D_mean _to the esophagus). These differences between the IMRT and SA/PA-VMAT plans are not statistically significant respectively (all *p *> 0.05).

Comparing to the IMRT plans (13.7 ± 2.6 minutes), the SA/PA-VMAT plans reduce the treatment time (10.6 ± 1.8 and 6.4 ± 1.5 minutes, respectively) significantly as expected (all *p *< 0.01). Also, we found that the SA/PA-VMAT techniques are more efficient in dose delivery during treatment than that of the IMRT plans (439.2 ± 22.5/435.7 ± 25.3 *vs*. 458.3 ± 21.9 MUs) (shown in Table 4).

**Table 4:** Comparisons of other evaluated parameters in present study (n = 12).

	IMRT	SA-VMAT		PA-VMAT		
	mean ± SD	mean ± SD	*p *value*^a^*	mean ± SD	*p *value*^b^*	*p *value*^c^*
Delivery time*^d^*	13.7 ± 2.6	10.6 ± 1.8	< 0.01	6.4 ± 1.5	< 0.01	< 0.01
Treatment Efficiency*^e^*	458.3 ± 21.9	439.2 ± 22.5	0.030	435.7 ± 25.3	0.047	0.732

*^a^*: compared with the parameters of IMRT; *^b^*: compared with the parameters of IMRT; *^c^*: compared with the parameters of SA-VMAT; *^d^*: presented as minutes; *^e^*: presented as monitor units (MUs);

## Discussion

As mentioned earlier, the implementation of the VMAT technique gains its value in the treatment for different kinds of the solid tumors, even the tumor targets shaped complex, compared to the conformal radiation therapy (CRT) and IMRT. In present study, we provided in details the dosimetric differences between IMRT and the SA/PA-VMAT plans in the treatment for locally advanced lung cancer.

At present, with the introduction of involved-field radiation therapy (IFRT) and omission of elective nodal irradiation (ENI) in treatment for locally advanced NSCLC, the PTVs of stage III cases, especially IIIB (involving the bilateral mediastinum) are somewhat complex and more like a "dumb bell". Referring to the suggestion from Guckenberger *et al*. [11], the SA-VMAT plans were generated and compard to IMRT plans. Under the consideration of reducing more treatment time, we also designed the case-individualized PA-VMAT plans for evaluation according to the location of the targets. As expected, our data indicate that in treatment planning for locally advanced lung cancer, a single arc VMAT plan achieve superior dose covarage for PTV (the CI and HI are all better, *p *< 0.05), even a partial arc VMAT could achieve such dose distribution compared to IMRT plans. This was also in a line with the study by Scorsetti *et al*. [3], the VMAT technique allowed them to achieve the most objectives on target volumes and OARs in radiation therapy for stage III NSCLC in practice. Recently, Yang *et al*. designed and generated a trajectory-based, noncoplanar subarc for VMAT delivery [14]. They found that after the plan optimization, the trajectory-based VMAT technique showed improved target dose conformality and reduced irradiation dose for OARs, compared to the standard VMAT and IMRT in treating central nervous system tumors. And the potential advantages of such technique in radiotherapy for NSCLC need further dosimetric evaluation.

The radiation-induced pneumonitis (RIP) is one of the most common side-effects in CRT for thoracic malignancies. This complication, which has a considerable impact on patient morbidity and sometime leads to death, is strongly correlated with the irradiation dose delivered to the lungs. A number of studies have indicated that the dosimetric parameters from the lung DVH are important in predicting RIP risk [15-18]. Several studies showed that MLD approach seems to provide the most consistent results in terms of increasing RIP rate with increasing MLD, but some stated not [15,16]. And many investigations indicated that the V_20 _and V_30 _also were predictive factors of RIP, especially of the severe grade (≥ 2) [17,18]. Our study found that the V_20_, V_30 _and MLD of the total and contralateral lungs were reduced significantly in the SA/PA-VMAT plans than those in IMRT plans respectively (*p *< 0.05, as shown in table 3). This founding is correlated with the study of McGrath *et al*. among lung cancer patients [4], and indicats the possible dosimetric advantages of VMAT in treatment for central and bulky lung cancers. Furthermore, we noticed that the PA-VMAT technique results in significantly better V_20_, V_30 _and MLD for the contralateral lung when compared to the SA-VMAT technique; while the total lung and ipsilateral lung parameters were not found to be different. Although we realized that SBRT regimens could not easily be compared with conventional CRT regimens, this findings is still partly correlated the results from Holt *et al*. [6], indicating the potential value of the PA-VMAT technique in treatment for the locally advanced lung cancers.

On the other hand, we noticed that the V_5 _and V_10 _of the total and contralateral lungs in the SA/PA-VMAT plans were higher than those of IMRT plans, respectively (*p *< 0.05). According to the Schallenkamp's report of a 92-patient cohort, the V_10/13/15 _was also significantly correlated to the RIP either [19]. Wang *et al*. found that V_5 _of both lung lobes was the only parameter predicting the RIP (≥ grade 2) in NSCLC patients treated with definitive concurrent chemoradiotherapy [20]. In a study from Netherlands, Palma *et al*. reported a case of the "Severe'' radiological pneumonitis 3 months after SBRT using VMAT and its corresponding treatment plan [21]. Although they concluded that severity and patterns of the RIP were similar, the VMAT techniques still obviously delivered low irradiation dose to a larger volume of lung than IMRT did. From this point of view, the VMAT technique might increase the RIP rate more than the IMRT dose.

The DVH-based parameters of the ipsilateral lung were similar in the IMRT and VMAT plans as displayed in table 3. We could not add the V_5-15 _and V_20/30 _of the ipsilateral lung as the predictive factors for the RIP following the investigations from Yorke *et al*. and Ramella *et al*. [22,23]. As it reduced middle dose volume but increasing low dose volume of the total and contralateral lungs, the conclusion could not be easily obtained that the SA/PA-VMAT techniques had the dosimetric advantages for locally advanced lung cancer from the present study.

In our study, we did not find significant differences in dose sparing of other OARs (spinal cord, heart and esophagus) between the IMRT and SA/PA-VMAT plans in treatment for locally advanced lung cancers.

Beside the dosimetric analysis, similar with other studies mentioned above [3,4,6,9-11], less treatment time was observed in plans delivering using the SA/PA-VMAT techniques. This would be another advantage of the VMAT technique: enhancing patient satisfaction and comfort and reducing intrafraction variation. It might potentially help patients tolerate a whole treament procedure, particularly those sick or painful cases. Also in a line with other studies, we observed that the SA/PA-VMAT plans achieved the comparable dose distribution of the targets with more less MUs, compared to IMRT technique (as shown in table 4).

To our knowdge, the applications of several other techniques that might be helpful to reduce the RIP in radiotherapy for locally advanced lung cancer, including the set-up correction with cone beam CT scan and the active breathing control during simulation and treatment. In present study, we only indicated the dosimetric advantages and disadvantages of the VMAT techniques in treatment for locally advanced lung cancers, compared with the IMRT technique. Even only 18% grade 1 and 9% grade 2 acute RIP were observed in 24 patients with large-volume NSCLC after VMAT treatment [3], the potential value of the VMAT techniques for central and bulky lung cancers needs more clinical investigations.

## Conclusions

In this dosimetric and efficiency analysis, the SA-VMAT plans show more optimal target coverage. Compared to the IMRT plans, the VMAT plans reduce the V_20/30 _and MLD but increase the V_5/10 _of the total and contralateral lungs. The PA-VMAT technique shows more lung dose sparing of the contralateral lung. With more efficiency, VMAT plans reduce the treatment time significantly. But studies are warranted to evaluate the clinical benefits of the VMAT in treatment for patients with locally advanced NSCLC in future.

## Conflict of interest notification

The authors declare that they have no competing interests. While our radiation physics center is a training center for Chinese radiation oncologists and physicists supported by Elekta Oncology Systems (Crawley, UK).

## Authors' contributions

XJ, TL and YG contributed equally in design of the study, collection of data and drafting the manuscript; YL, LZ, YX and XZ worked on collection of data and critical revision of the manuscript; YG provided the conception of this study and the final approval of the version to be published. And all authors read and approved the final version of the manuscript.
